# Pes Anserinus Bursitis due to Tibial Spurs in Children

**DOI:** 10.7759/cureus.1427

**Published:** 2017-07-05

**Authors:** Vivek Tiwari, Venkatesan Sampath Kumar, Rishi R Poudel, Ashok Kumar, Shah Alam Khan

**Affiliations:** 1 Department of Orthopaedics, All India Institute of Medical Sciences, New Delhi, India

**Keywords:** pes anserinus, osteochondroma, tibial spur, rose thorn, exostosis

## Abstract

Osteochondromas are the most common bone tumours. Although these tumors are relatively common in the long bones of children, the varied clinical and radiographic presentation of such neoplasms around the knee joint can cause diagnostic delays, especially when not associated with a palpable swelling. Proximal tibial osteochondromas can sometimes unusually present as spurs/ rose thorns leading to pes anserinus bursitis and vague knee pain. We describe the clinico-radiographic features of such proximal tibial metaphyseal osteochondromas giving rise to pes anserinus bursitis in three children, including bilaterally symmetrical osteochondroma in one of the cases, who were treated conservatively with good outcomes.

## Introduction

Knee pain in children can have varied causes both innocuous and sinister. Osteochondroma represents the most common bone tumor and is a developmental lesion rather than a true neoplasm. It constitutes 20%-50% of all benign bone tumors and 10%-15% of all bone tumors [1]. Osteochondromas of the proximal tibia as a cause of knee pain in a child are not uncommon, although proximal tibial osteochondromas can have varied presentations ranging from knee pain to actual locking of the knee joint [2]. We present a report of three children with proximal tibial osteochondromas presenting as pes anserinus bursitis including bilaterally symmetrical tumors in one patient, with uncommon imaging features.

## Case presentation

Case 1

**Figure 1:**
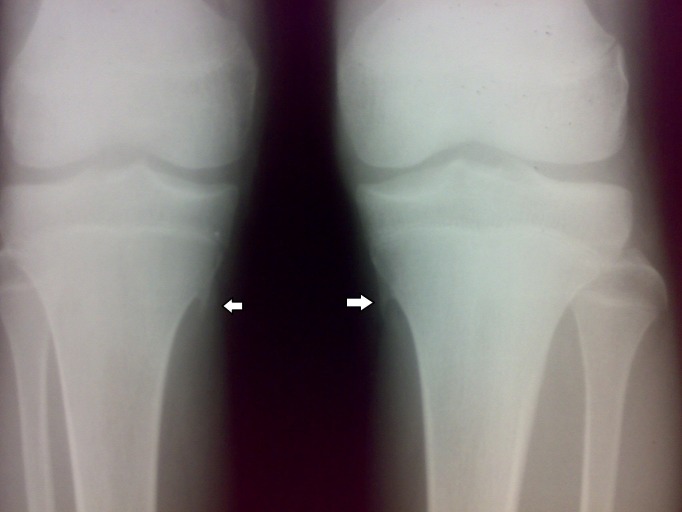
Case 1: Plain radiograph of bilateral knees—anteroposterior view Bilateral, symmetrical, sharp, bony spurs (white arrows) arising from the proximal tibial metaphysis corresponding to attachment of the hamstring tendons.

**Figure 2:**
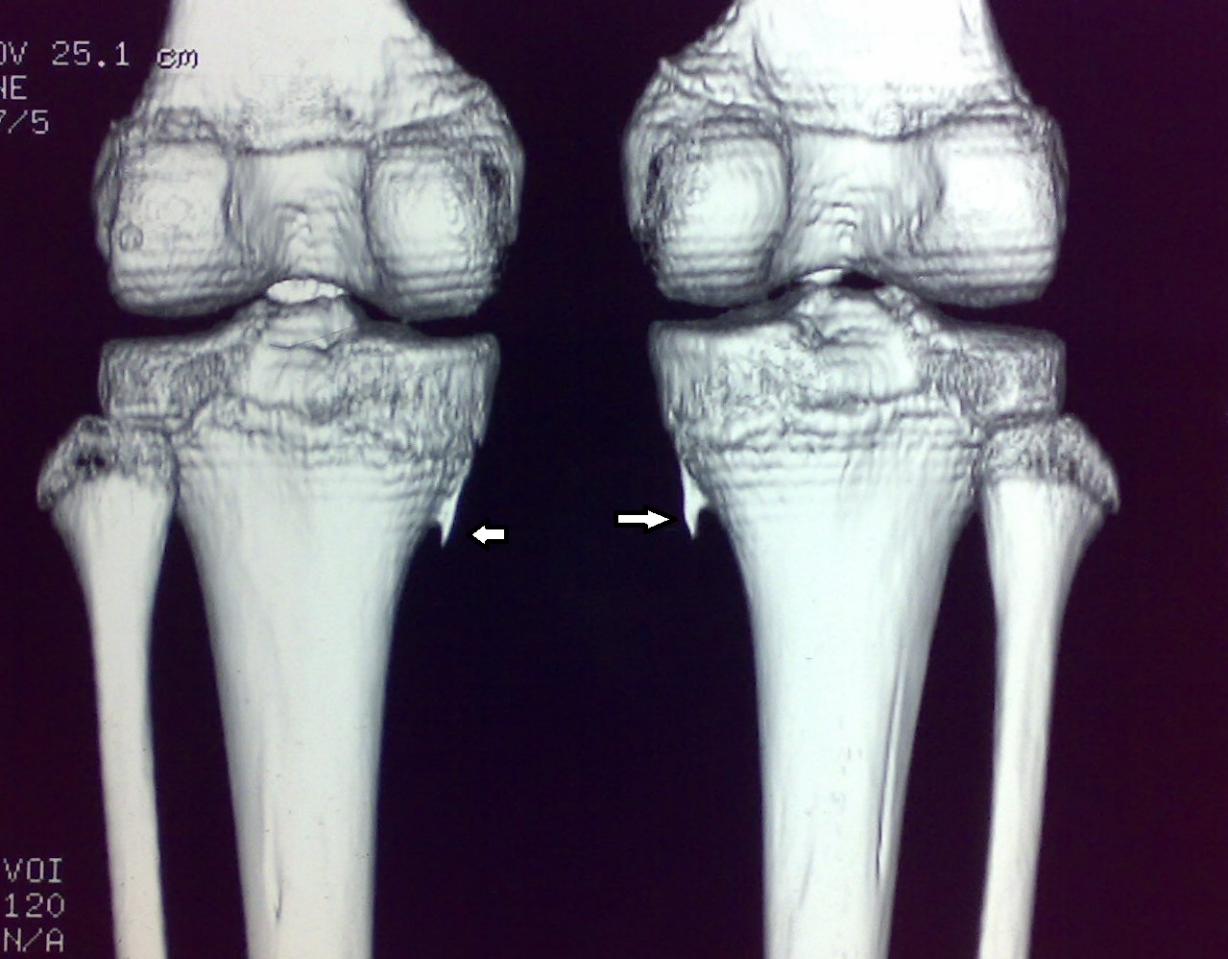
Case 1: Three-dimensional (3D) reconstruction CT scan of both knees Sharp “rose-thorn” like bony spurs (white arrows) arising from the medial proximal tibial metaphysis of both legs hanging down like an icicle.

A 10-year-old male child presented to us with pain over the medial aspect of both knees since the last two months. There was no history of trauma, fever, or pain in other joints. On clinical examination, it was found that there was localized tenderness over the medial aspect of the proximal tibia at the attachment of pes anserinus. Tenderness was marginally more with the knee joint in extension. Overlying skin was normal. No underlying bony swelling was palpable. The knee joint was stable. There were no bony swellings in other limbs. Systemic examination of the child was normal. Radiographic examination of both the knee joints revealed bilateral, symmetrical, sharp, bony spurs arising from the proximal tibial metaphysis corresponding to the attachment of the hamstring tendons (Figure 1). A computed tomography (CT) scan of both knees was done to further characterize the lesions. CT scans of both the knees showed sharp “rose-thorn” like bony spurs arising from the medial proximal tibial metaphysis hanging down like an icicle (Figure 2).  The spurs had no cap. A clinico-radiological diagnosis of bilateral symmetrical tibial osteochondroma with pes anserinus bursitis was made.

The child was initially managed by giving rest and routine analgesics for a period of three weeks. All sporting activities were restricted for the said period. There was an initial reduction in his symptomatology but pain reappeared following return to routine activities. Injections of hydrocortisone and 2% xylocaine were given bilaterally at the most tender point of the pes anserinus area followed by a period of rest for two weeks. The patient showed complete resolution of pain and was symptom free two years after injections.

Case 2

**Figure 3:**
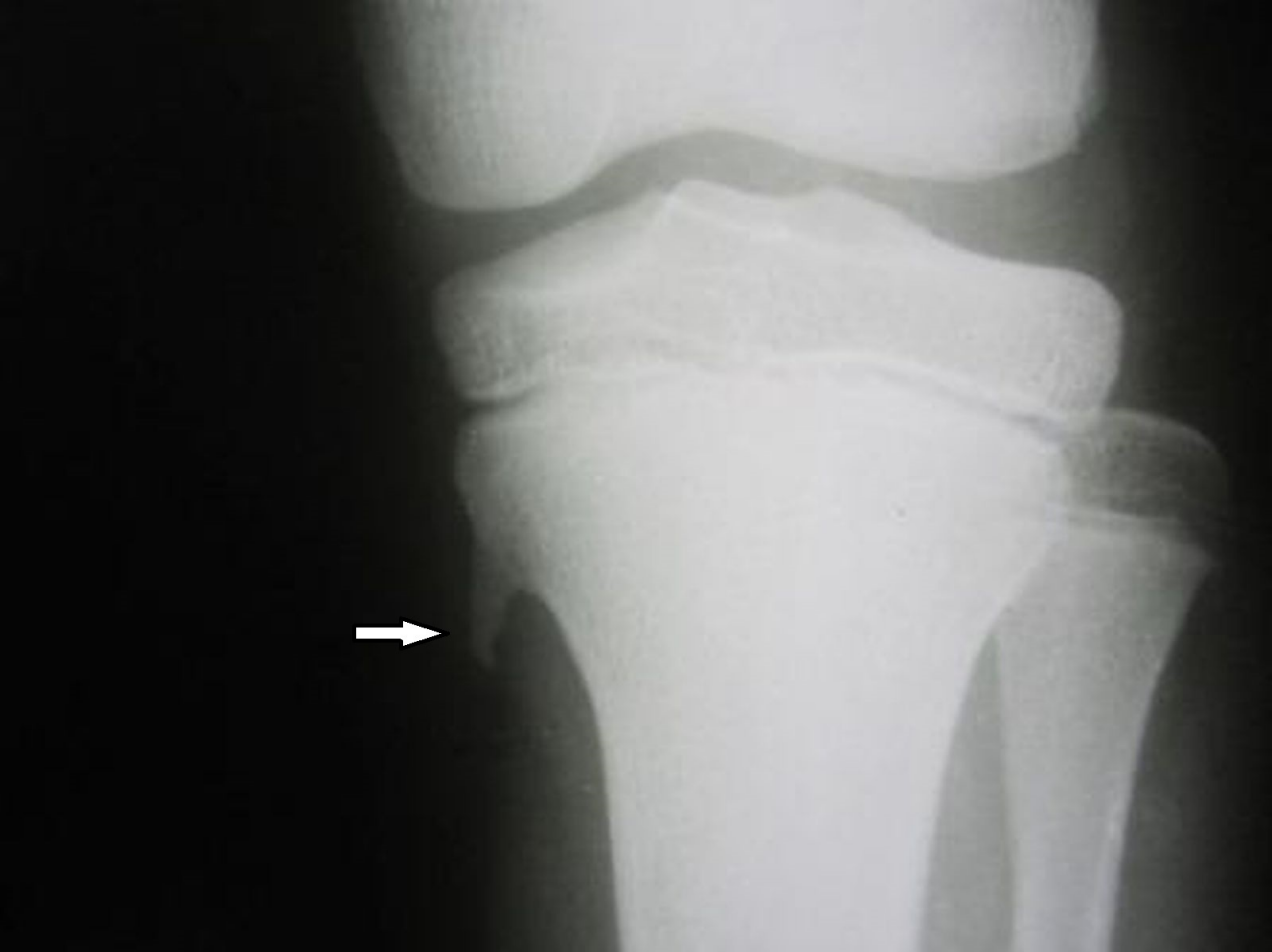
Case 2: Plain radiograph of the left knee—anteroposterior view Sharp bony spur (white arrow) arising from proximal tibial metaphysis, similar to the first case.

Another 13-year-old male child presented to the outpatient department with similar complaints of knee pain on the left side for the last two months. The onset was insidious and there was no history of trauma. There was no history of swelling or pain in any other part of the body. On examination, it was found that tenderness was present at the medial aspect of left proximal tibia. No swelling was palpable. Rest of the local examination and systemic examination were unremarkable. Plain radiographs of the left knee revealed a sharp bony spur arising from proximal tibial metaphysis, similar to the first case (Figure 3). But the radiograph of the contra-lateral knee was unremarkable. The patient was again advised rest and analgesic medications for three weeks. No other subsequent intervention was required, and at the last follow-up of two years, there was no recurrence of pain.

Case 3

**Figure 4:**
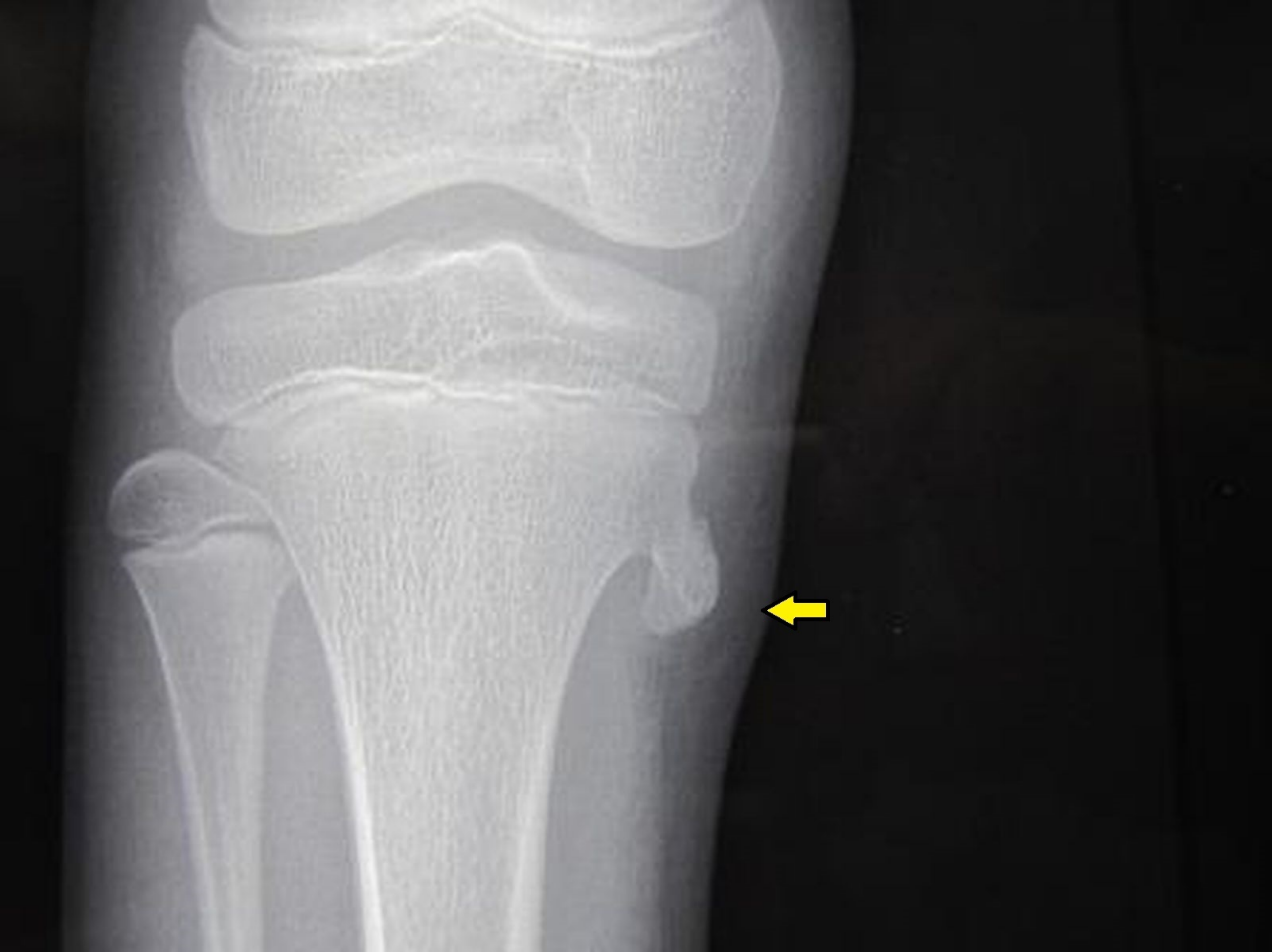
Case 3: Plain radiograph of the right knee—anteroposterior view Presence of a bony outgrowth (yellow arrow) from the medial aspect of right proximal tibial metaphysis suggestive of an osteochondroma.

The third child was a 12-year-old male presenting to the outpatient department with complaints of swelling located in the medial aspect of right knee for the last three months. The swelling was insidious in onset without any history of trauma or fever. It was of the size of a lemon, as described by the patient’s guardian. There was associated vague pain in the same area, without any aggravating factor. There was no history of rapid increase in the size of swelling or the intensity of pain. Similar to the other two cases, there was no other swelling or pain elsewhere in the body. On examination, it was found that there was a bony hard, spherical, nodular, tender swelling on the medial aspect of right proximal tibia measuring 3 x 3 cm in size. The overlying skin was normal. Range of motion of knee joint was comparable to the other side. Plain radiograph of the right knee showed the presence of a bony outgrowth from the medial aspect of right proximal tibial metaphysis suggestive of an osteochondroma (Figure 4). There was no evidence of malignant transformation on the plain radiograph. The patient was advised rest from exertional activities along with analgesic medications for three weeks causing reduction in pain. However, soon the pain started again gradually over a few days, for which the patient was injected with injection hydrocortisone and 2% xylocaine at the most tender point in the pes anserinus area. After this intervention, the patient became pain-free. The patient was regularly followed-up to check malignant transformation of the swelling. At the last follow-up of one-and-a-half years, there was no increase in the size of swelling or recurrence of pain.

## Discussion

Osteochondromas, the most common bone tumours, can be seen in up to 3% of the general population [3]. The proximal tibial metaphysis around the area of pes anserinus insertion is one of the most common sites affected by solitary osteochondromas [4]. However, there is paucity of literature on the natural history, varied radiological presentations and management strategies of proximal tibial osteochondromas presenting as pes anserinus bursitis particularly in the paediatric population.

According to Ugai et al., exostoses occurring only in the pes anserinus may or may not be osteochondromata and should be classified as pes anserinus bony spurs [5]. Isolated tibial exostoses of the pes anserinus can be differentiated from exostoses in patients with multiple exostoses (osteochondromas) by an icicle appearance in roentgenography with no detectable cartilage cap, as was seen in our cases. On the other hand, exostoses of multiple osteochondromas show varying appearances in roentgenography with cartilage caps on their surfaces [5]. Such bony spurs are also called the “cloth hook exostosis” [6].

The imaging of these lesions is atypical but self-explanatory. The CT scan delineates the 3D morphology of the spur and is essential before attempting its surgical excision. Both CT and magnetic resonance imaging (MRI) scans are helpful in showing the cartilage cap over the spur although an MRI is a better imaging tool to assess the cartilage cap thickness [7].

The treatment of tibial bony spurs is dependent on the location and severity of symptoms. The spurs need not be excised if the symptoms improve with rest and do not recur following resumption of activities. Surgical treatment is limited to patients with intractable, recalcitrant pain [8]. However, surgeons need to be aware of such unusual presentation of osteochondromas, especially in children. To the best of our knowledge, pediatric cases with such unusual radiological presentation of osteochondroma leading to pes anserinus bursitis have been sparingly described in literature. Suspicion of such lesions in pediatric cases with knee pain will help in prompt identification and timely diagnosis thereby decreasing overall morbidity.

## Conclusions

Osteochondromas of the proximal tibial metaphysis can sometimes present as a “rose thorn” or a spur, which in children can rarely give rise to irritation of the pes anserinus bursa. After identification and proper evaluation, such patients should be adequately treated for the pes anserinus bursitis, besides monitoring for a possible malignant transformation. Most of such cases can be managed conservatively; surgical treatment may be rarely required in recalcitrant cases.
